# The Nuffield Sponsorship Ends

**Published:** 1991-12

**Authors:** 


### The Nuffield Sponsorship Ends

In 1989 Nuffield Hospitals Trusts gave the Bristol Medico-Chirurgical Journal an extremely generous sponsorship, enabling it in 1990 to carry out a longstanding ambition — to transform itself into a journal genuinely serving the whole of the West of England. This sponsorship has continued through 1991 and will end in mid 1992. The formation of the West of England Medical Journal Association would not have been possible without the Nuffield Sponsorship and for this generous, and we would say enlightened gesture, the medical fraternity in the West of England gives them sincere thanks. Since 1986 our journals have been circulated free to West of England doctors through sponsorship first of Hermiston with Bristol-Myers and Pfizer, then Clinical Press and finally Nuffield. We shall seek further sponsorship but we may not find it. Will the societies forming the Association now be prepared to fund their own journal? The cost spread among them is relatively trivial, to each individual perhaps the cost of 3 gallons of petrol a year, which for most is tax deductable as part of the subscription to their society or club. If they will find this the journal can continue, if not then perhaps one must conclude indifference to whether it survives or not. Doctors have become so used to being courted by sponsors that paying for anything seems to go against the grain. Could the West of England Medical Journal be the exception?

